# Prevalence and impacts of breathlessness in South Africa: an exploratory, cross-sectional population study

**DOI:** 10.7189/jogh.16.04229

**Published:** 2026-10-09

**Authors:** Slavica Kochovska, Vanessa N Brunelli, Sungwon Chang, Irina Kinchin, Danny J Eckert, Joseph D Clark, Jacob Sandberg, Magnus Ekström, David C Currow, Richard N van Zyl-Smit

**Affiliations:** 1 College of Medicine and Public Health, Flinders University, Adelaide, Australia; 2 Faculty of Health, University of Technology Sydney, Sydney, Australia; 3 Faculty of Science, Medicine and Health, University of Wollongong, Wollongong, Australia; 4 Centre for Health Policy and Management, Discipline of Public Health and Primary Care, School of Medicine, Trinity College Dublin, University of Dublin, Dublin, Ireland; 5 Wolfson Palliative Care Research Centre, University of Hull, Hull, UK; 6 Respiratory Medicine, Allergology and Palliative Medicine, Department of Clinical Sciences Lund, Lund University, Lund, Sweden; 7 Department of Health, Blekinge Institute of Technology, Karlskrona, Sweden; 8 Division of Pulmonology and UCT Lung Institute, Department of Medicine, University of Cape Town and Groote Schuur Hospital, Cape Town, South Africa

**Keywords:** breathlessness, cross-sectional survey, population study, higher-middle income, quality of life, South Africa

## Abstract

**Background:**

Chronic breathlessness contributes substantially to the global health burden and healthcare use, yet data on its prevalence and impacts in lower-resource settings remain limited. We investigated the prevalence, self-reported underlying conditions, and impacts of breathlessness at the population level in South Africa.

**Methods:**

We conducted a cross-sectional online survey in English among community-dwelling adults aged 18 years or older. The sample was stratified by age, sex, and rurality to reflect the 2022 National Census. Self-reported data included sociodemographic characteristics; breathlessness, assessed using the modified Medical Research Council (mMRC) breathlessness scale; employment; activities of daily living, assessed using an item from the London Chest Activities of Daily Living; quality of life, assessed using the EQ-5D-5L and EQ visual analogue scale; and disability, assessed using the 12-item World Health Organization Disability Assessment Schedule 2.0. We analysed the weighted dataset using descriptive statistics.

**Results:**

We included 2,828 respondents with a mean age of 37.1 years (standard deviation = 12.4), of whom 52.3% were female. Clinically important breathlessness (mMRC ≥ 2) was reported by 189 respondents (6.7%), with a median duration of 3.1 years (interquartile range = 1.5–5.4). The most commonly reported attributions were lung conditions other than tuberculosis (23.8%), ‘other’ factors (16.4%), and anaemia (11.6%). Clinically important breathlessness was associated with poorer perceived health, lower quality of life, and higher total disability scores than mild (mMRC 1) or no breathlessness (mMRC 0). Mobility and participation were the most affected disability domains. Among respondents with an mMRC≥2, 64 (33.8%) reported an impact on employment, of whom 49 (76.6%) had ceased full-time work, while 171 (90.3%) respondents reported an impact on daily activities.

**Conclusions:**

We estimate that more than four million people in South Africa live with clinically important breathlessness. The symptom is associated with poorer quality of life, greater disability, reduced employment, and impaired function. The life-changing effects of this debilitating symptom among community-dwelling adults should be investigated in longitudinal studies to help inform clinical policy and practice.

Global health policy, research, and practice primarily focus on diseases and their risk factors, with less attention given to the chronic symptoms that many diseases generate [1]. However, symptoms can also contribute substantially to the health burden and healthcare use, particularly in the context of chronic or progressive illnesses. For this reason, understanding the population-level prevalence, severity, and impacts of symptoms is critical for identifying the magnitude of health-related disability across communities and developing effective, evidence-based interventions to improve individual and societal outcomes.

Chronic breathlessness limiting exertion (hereafter referred to as ‘breathlessness’) is a cardinal symptom of many chronic conditions [2] and a major contributor to the global health burden. Approximately 10% of adults in high-income countries live with clinically important breathlessness [3,4], which is defined as a score of ≥2 on the modified Medical Research Council (mMRC) breathlessness scale: ‘I walk slower than people of the same age on the level because of breathlessness or have to stop for breath when walking at my own pace on the level’ or worse [5]. While its prevalence in lower- and middle-income settings is largely unknown, one study from India found that 20% of adults lived with clinically important breathlessness [6].

Irrespective of geographical setting, breathlessness is associated with poorer quality of life, limitations in everyday activities, disability, and adverse economic outcomes [3,6]. However, the magnitude of these impacts may differ between higher- and lower-resource settings because of socioeconomic, cultural, and health-related factors. Compared with high-income countries, lower- and middle-income countries have higher rates of communicable and non-communicable diseases, such as tuberculosis (TB) and chronic obstructive pulmonary disease (COPD), as well as a higher prevalence of risk factors associated with breathlessness, including tobacco smoking and air pollution [6–8]. This highlights the need for research on breathlessness across different socioeconomic and geographical contexts. Simultaneously, the epidemiology of the condition should be investigated independently of healthcare contact, as access to health services may vary among people living with breathlessness [9]. Such studies would provide a better understanding of its health-related burden and outcomes.

The Republic of South Africa is one example of an upper-middle-income country with a high burden of diseases commonly associated with breathlessness [10,11]. TB is a leading cause of morbidity and mortality, particularly among people living with HIV/AIDS, with high rates of HIV-associated and drug-resistant TB [11–13]. Post-TB lung disease is also increasingly recognised among people who survive TB, but experience long-term respiratory symptoms [14]. Another leading cause of morbidity and mortality is COPD [15], which is prevalent among smokers and people exposed to second-hand tobacco smoke or biomass fuel smoke from indoor cooking, particularly in rural areas [16]. Asthma prevalence is also high [11]. Together, the high prevalence of these conditions and risk factors are likely to contribute substantially to breathlessness in the South African population [17]. A better understanding of the prevalence of breathlessness, its potential causes, and its impacts is needed to inform healthcare, policy, and clinical practice.

Therefore, we aimed to investigate the prevalence, self-reported underlying conditions, and impacts of breathlessness at the population level in South Africa.

## METHODS

South Africa is an upper-middle-income country with a population of 63.2 million people as of 2023 [18]. Most people live in urban areas (66%), and just over half of the population (56%) lives in three provinces – Gauteng, KwaZulu-Natal, and the Western Cape [18]. English, one of the country’s 12 official languages, serves as the *lingua franca*, with 48.4% of the population (31 million of 64 million people) estimated to speak and understand the language, while 9% speak only English [19,20]. South Africa has high internet and mobile phone penetration, with 45.3 million internet users (75% of the population) and 118.6 million active mobile subscriptions. This corresponds to 195 subscriptions per 100 people and indicates that many people have more than one subscription, such as for a secondary phone, tablet, or other connected device [21]. We reported the study according to the Strengthening the Reporting of Observational Studies in Epidemiology (STROBE) guidelines [22].

### Study design

We conducted a cross-sectional online survey in English among community-dwelling adults aged 18 years or older in South Africa. We used a predetermined sample size of 3000 participants, based on similar population-based surveys conducted in lower-middle- and high-income countries [6,23]. We stratified recruitment by age, sex, and rurality to obtain a sample broadly reflective of the 2022 South African Census [18].

We conducted recruitment through a market research company Qualtrics (Provo, Utah, USA), whereby we designed the study questionnaire, while Qualtrics randomly distributed it across multiple panels that comprised registered members who had consented to participate in survey research. The use of several panels helped create a blended, nationally representative sample and reduce the risk of bias associated with any single panel. Qualtrics complies with International Organization for Standardization certification requirements [24,25], including requirements related to equitable operations in South Africa. Panel members received USD 2.00–3.50 per survey as compensation for their time, with rates determined according to fair market value based on the time spent completing any given survey and the proportion of eligible participants who completed it.

Potential participants received e-mail invitations containing an embedded link to the survey on the Qualtrics platform, which could be completed on a computer, mobile phone, or tablet within 10–15 minutes. Before survey entry, a participant information sheet (PIS) describing the study was made available to participants on the survey’s landing page. The PIS, with permission from the relevant human ethics research committees, stated that the survey concerned respondents’ health, without specifically mentioning breathlessness. Potential participants then provided informed consent to participate and to allow deidentified, aggregated data to be used in future research. Qualtrics checked each respondent’s internet protocol address and used fingerprinting and deduplication technologies to ensure data integrity and prevent duplicate responses.

### Eligibility

Eligible participants were community-dwelling adults aged 18 years or older living in South Africa who had consented to participate in research through the market research company. No additional exclusion criteria were applied.

### Survey development and piloting

The survey used validated tools where possible, together with additional questions contextualised to South Africa based on advice from a resident clinical researcher (RNvZS). These measures helped ensure that the questions were culturally sensitive and relevant to the South African population. We piloted the survey on 31 May 2023 among 50 Qualtrics panel members in South Africa to assess question feasibility, cognitive interpretation, and survey usability. These responses were excluded from the main survey. No changes were made following the pilot. We conducted the full survey from 31 May to 20 July 2023.

### Assessments

Data collection relied on self-reporting. Demographic characteristics included age, sex, place of residence (urban *vs* rural; multiple-choice response regarding the province in which the person usually lives), highest level of education (multiple-choice response), smoking (current status for tobacco and, separately, cannabis smoking; and second-hand smoking for both substances) and employment (multiple-choice response regarding current status).

Breathlessness was assessed using the mMRC breathlessness scale [5]. Used extensively in population research, this five-point ordinal scale ranges from 0 to 4 and measures the level at which breathlessness limits exertion. Scores range from 0 (‘I only get breathless with strenuous exercise’) to 4 (‘I am too breathless to leave the house or when dressing or undressing’), with higher scores indicating a greater impact on daily functioning.

Respondents with an mMRC score of 1 or higher were asked about:

1) the duration of their breathlessness at the reported mMRC level, recorded in months or years as free text;

2) the underlying condition to which they primarily attributed their breathlessness, using the question ‘What do you understand is causing your breathlessness?’. Respondents selected one primary condition from a multiple-choice list, which included ‘don’t know’ and an option to provide free text if they selected ‘other’;

3) the effect of breathlessness on their activities of daily living, using a single item from the London Chest Activities of Daily Living questionnaire [26], with the response options being ‘a lot’, ‘a little’, and ‘not at all’;

4) whether breathlessness had affected their ability to work. Those who responded ‘yes’ selected their employment status before breathlessness affected their ability to work from a multiple-choice list.

The mMRC and breathlessness-related assessment are provided in Documents S1–5 in the **Online Supplementary Document**.

The EuroQol EQ-5D-5L descriptive system and visual analogue scale were used to assess health-related quality of life. EQ-5D-5L comprises five dimensions: mobility, self-care, usual activity, pain or discomfort, and anxiety or depression. Each dimension is rated across five levels of severity: no problems, slight problems, moderate problems, severe problems, or extreme problems. The EuroQol visual analogue scale (EQ-VAS) assesses current health status from 0 to 100, representing the worst and best health, respectively [27,28].

The 12-item World Health Organization Disability Assessment Schedule 2.0 (WHODAS) was used to assess disability across six life domains: cognition, mobility, self-care, getting along, life activities, and participation [29]. Each domain comprises two items, with each item scored on a scale from 0 (no difficulty) to 4 (extreme difficulty). Domain scores range from 0 to 8, while total disability scores range from 0 to 48. Three additional questions within the tool assess the extent to which these difficulties affect individuals’ lives. We used the total score for statistical analysis and graphed each domain to identify which aspects of life were most impaired. The WHODAS is a standardised measure of health and disability across conditions, populations, and cultures [29].

### Statistical analysis

We tabulated sociodemographic characteristics by mMRC score and calculated frequency distributions for categorical variables. We assessed differences between groups using Pearson χ^2^ test for categorical variables and the Kruskal-Wallis test for continuous variables.

We defined clinically important breathlessness as an mMRC score of 2 or higher, corresponding to ‘I walk slower than people of the same age on the level because of breathlessness or have to stop for breath when walking at my own pace on the level’, or worse. Mild breathlessness was defined as an mMRC score of 1, corresponding to ‘I get short of breath when hurrying on the level or walking up a slight hill’. We quantified mild breathlessness separately to provide a more complete understanding of the presence and impact of breathlessness in the community, given its associations with disability [30], poorer quality of life [31], increased healthcare use and hospital admissions, and longer inpatient stays [32–34]. We combined mMRC scores of 3 and 4 for the analyses because of the small number of responses with an mMRC score of 4.

We applied calibration weighting using iterative proportional fitting (raking) to align the sample with population benchmarks from the 2022 Statistics South Africa Census [17] to improve the representativeness of the sample. We only included participants with complete demographic data required to construct the weights. We selected calibration variables *a priori* based on data availability, complete sample support, and established associations with health outcomes. These variables included age, sex, place of residence (urban or rural), and highest educational attainment, categorised as some secondary education, completed secondary education, and post-secondary education in accordance with Census classifications.

We assigned higher weights to under-represented groups and lower weights to over-represented groups relative to the Census distributions during the raking procedure. Educational attainment remained somewhat higher than the population benchmarks after weighting, indicating residual imbalance. We did not include population group (race), income, employment sector, or province in the weighting, due to their unavailability in the survey, incompatibility with the analytic sample (*e.g.* employment sector among employed participants), or sparse or zero cell counts that precluded stable calibration.

In the unweighted data set, missing data were minimal: four participants had missing data for sex, and seven had missing data for smoking status, recorded as ‘prefer not to respond’. No data were missing for breathlessness (measured using the mMRC scale), the primary outcomes (measured using the EQ-5D-5L, EQ-VAS, and WHODAS), or the key demographic covariates. We conducted complete-case analyses without imputation because the proportion of missing data was low (<5%) [35].

All analyses accounted for the complex survey design using the ‘Complex Samples’ module in SPSS. We applied calibration weights to generate population-representative estimates and used Taylor series linearisation to estimate standard errors and 95% confidence intervals (CIs).

We estimated the prevalence of moderate-to-severe breathlessness, defined as an mMRC score of 2–4, using survey-weighted proportions. We estimated adjusted prevalence using multivariable logistic regression, with mMRC category (scores from 2–4 compared with 0 –1) as the dependent variable and age, sex, smoking status, place of residence, and educational attainment as covariates. We calculated predicted probabilities for each participant and averaged them across the analytic sample to obtain adjusted prevalence estimates with 95% CIs.

Continuous outcomes were quality of life, measured using the EQ-VAS, and functional disability, measured using the 12-item World Health Organization Disability Assessment Schedule (WHODAS 2.0; range = 0–48) [28,29]. We calculated means and standard deviations and examined associations with covariates using survey-weighted linear regression models. We estimated adjusted mean EQ-VAS and WHODAS-12 scores across mMRC categories of 0, 1, 2, and 3–4 using model-based marginal means. While the EQ-VAS scores were somewhat skewed, we considered linear regression appropriate because of the large sample size and its robustness to moderate deviations from normality.

Variables used to construct the survey weights were age, sex, education, and place of residence. We also included these variables as covariates in the regression models to improve precision and reduce potential residual confounding related to the sampling design. We conducted sensitivity analyses to assess residual imbalance and overlap-restricted strata. We conducted all statistical analyses using SPSS, version 28.0 (IBM Corp., Armonk, New York, USA).

## RESULTS

The weighted analytic sample included 2828 respondents (**Table 1**) with a mean age of 37.1 years (standard deviation (SD) = 12.4), of whom 52.3% were female. Most respondents lived in urban areas (68.3%), with the largest proportion of respondents living in Gauteng province (20.9%). More than half of the respondents had completed grade 12 (52.7%). Overall, 43.9% had never smoked tobacco, while 58.6% had never smoked cannabis.

**Table 1:** Characteristics of a population sample of adults in South Africa by breathlessness level per the mMRC categories, weighted data*

	0 (n = 1,620, 57.3%)	1 (n = 1,019, 36.0%)	2 (n = 138, 4.9%)	3–4 (n = 50, 1.8%)	Total (n = 2,828, 100%)
**Age**					
x̄ (SD)	36.9 (13.0)	37.1 (13.1)	38.4 (13.3)	37.1 (12.4)	37.1 (13.0)
Mdn (IQR)	36.0 (27.0– 49.0)	38.0 (28.0–52.0)	39.0 (31.0–55.0)	35.9 (26.0–51.0)	37.0 (27.0–51.0)
**Sex**†					
Male	856 (52.8)	414 (40.6)	57 (41.0)	22 (44.0)	1,349 (47.7)
Female	764 (47.2)	605 (59.4)	82 (59.0)	28 (56.0)	1,479 (52.3)
**Place of residence**					
Urban	1,083 (66.9)	708 (69.5)	106 (76.3)	35 (70.0)	1,932 (68.3)
Rural	537 (33.1)	310 (30.4)	33 (23.7)	15 (30.0)	895 (31.6)
**Province ^~^**					
Western Cape	177 (10.9)	140 (13.7)	24 (17.3)	4 (8.0)	345 (12.2)
Free State	240 (14.8)	114 (11.2)	14 (10.1)	6 (12.0)	374 (13.2)
Kwa-Zulu Natal	46 (2.8)	21 (2.1)	2 (1.4)	1 (2.0)	70 (2.5)
North West	89 (5.5)	40 (3.9)	7 (5.0)	1 (2.0)	137 (4.8)
Gauteng	327 (20.2)	213 (20.9)	26 (18.7)	24 (48.0)	590 (20.9)
Mpumalanga	97 (6.0)	72 (7.1)	8 (5.8)	4 (8.0)	181 (6.4)
Limpopo	348 (21.5)	262 (25.7)	34 (24.5)	7 (14.0)	651 (23)
Outside South Africa	120 (7.4)	78 (7.7)	7 (5.0)	1 (2.0)	206 (7.3)
Do not know	174 (10.7)	80 (7.9)	17 (12.2)	3 (6.0)	274 (9.7)
**Education ^~^**					
No formal education	5 (0.3)	8 (0.8)	0 (0.0)	0 (0.0)	13 (0.5)
Less than 10th grade	1 (0.1)	0 (0.0)	0 (0.0)	4 (8.0)	5 (0.2)
10th grade completed	45 (2.8)	20 (2.0)	3 (2.2)	0 (0.0)	68 (2.4)
12th grade completed	851 (52.5)	543 (53.3)	71 (51.1)	24 (48.0)	1,489 (52.7)
College/University completed	612 (37.8)	384 (37.7)	54 (38.8)	17 (34.0)	1,067 (37.7)
Postgraduate degree completed	107 (6.6)	63 (6.2)	11 (7.9)	5 (10.0)	186 (6.6)
**Smoking status – Tobacco**					
Current smoker	472 (29.1)	335 (32.9)	61 (43.9)	4 (8.0)	887 (31.4)
Former smoker	366 (22.6)	269 (26.4)	35 (25.2)	0 (0.0)	684 (24.2)
Never smoked	775 (47.8)	409 (40.1)	42 (30.2)	24 (48.0)	1,241 (43.9)
Prefer not to say	7 (0.4)	5 (0.5)	1 (0.7)	17 (34.0)	16 (0.6)
**Smoking status – Cannabis**					
Current smoker	263 (16.2)	171 (16.8)	40 (28.8)	10 (2.0)	484 (17.1)
Former smoker	336 (20.7)	262 (25.7)	41 (29.5)	16 (3.2)	655 (23.2)
Never smoked	1,003 (61.9)	579 (56.8)	57 (41.0)	19 (3.8)	1,658 (58.6)
Prefer not to say	19 (1.2)	6 (0.6)	1 (0.7)	5 (1.0)	31 (1.1)
**Does anyone in your household currently smoke (tobacco and/or cannabis)**					
Yes	815 (50.3)	574 (56.3)	97 (69.8)	37 (7.4)	1,523 (53.9)
No	805 (49.7)	445 (43.7)	42 (30.2)	13 (2.6)	1,305 (46.1)
**Employment**					
Work full-time	758 (46.8)	476 (46.7)	71 (51.1)	27 (54.0)	1,332 (47.1)
Work part-time	239 (14.8)	137 (13.4)	22 (15.8)	8 (16.0)	406 (14.4)
Work on a casual basis	80 (4.9)	43 (4.2)	3 (2.2)	4 (8.0)	130 (4.6)
Retired/on pension	128 (7.9)	105 (10.3)	17 (12.2)	0 (0.0)	250 (8.8)
Unable to work because I cannot find a job	256 (15.8)	139 (13.6)	12 (8.6)	6 (12.0)	413 (14.6)
Unable to work because of disability	4 (0.2)	21 (2.1)	5 (3.6)	1 (2.0)	31 (1.1)
Other	114 (7.0)	75 (7.4)	9 (6.5)	3 (6.0)	201 (7.1)
Prefer not to say	15 (0.9)	6 (0.6)	0 (0.0)	1 (2.0)	22 (0.8)
Not applicable	25 (1.5)	16 (1.6)	0 (0.0)	0 (0.0)	41 (1.4)
**Breathlessness duration, years‡**					
Mdn (IQR)	NA	2.3 (1.3–5.0)	3.2 (1.5–5.4)	2.8 (1.2–5.5)	2.5 (1.3–5.0)
**Breathlessness underlying cause‡**					
Other§	NA	232 (22.8)	16 (11.5)	15 (30.0)	263 (21.8)
Do not know	NA	197 (19.3)	16 (11.5)	4 (8.0)	217 (18.0)
Lung conditions other than tuberculosis (*e.g.* emphysema, bronchitis, asthma, bronchiectasis)	NA	164 (16.1)	37 (26.6)	8 (16.0)	209 (17.3)
Poor nutrition	NA	135 (13.2)	15 (10.8)	6 (12.0)	156 (12.9)
Low blood count	NA	91 (8.9)	20 (14.4)	2 (4.0)	113 (9.4)
Heart conditions	NA	76 (7.5)	17 (12.2)	4 (8.0)	97 (8.0)
COVID	NA	47 (4.6)	6 (4.3)	2 (4.0)	55 (4.6)
HIV or AIDS	NA	25 (2.5)	3 (2.2)	4 (8.0)	32 (2.6)
Tuberculosis	NA	26 (2.5)	3 (2.2)	3 (6.0)	32 (2.6)
Disorders of the nerves or muscles	NA	22 (2.2)	3 (2.2)	1 (2.0)	26 (2.2)
Cancer	NA	4 (0.4)	1 (0.7)	1 (2.0)	6 (0.5)

IQR – interquartile range, Mdn – median, mMRC – modified Medical Research Council breathlessness scale, NA – not applicable, SD – standard deviation, x̄ – mean

*Presented as n (%) unless specified otherwise.

†Four cases were excluded from weighting due to respondents selecting ‘other’ or ‘preferred not to say’.

‡Questions on duration and primary underlying condition of breathlessness apply only to mMRC≥1 (n = 1,208).

§Other causes for the total population included: lack of fitness (n = 87), being overweight (n = 43), smoking (n = 51), anxiety/stress (n = 10), older age (n = 10), pregnancy-related (n = 6), and miscellaneous other (n = 55).

### Breathlessness prevalence

Clinically important breathlessness (mMRC ≥ 2) was reported by 189 of 2,828 (6.7%) respondents, and mild breathlessness (mMRC 1) by 1,019 of 2,828 (36.0%) respondents. The median duration of clinically important breathlessness at the reported mMRC level was 3.1 years (interquartile range = 1.5–5.4). Breathlessness was more commonly reported by women than men and by respondents living in urban rather than rural areas across mMRC scores 1–4 (**Table 1**).

In multivariable analyses, we estimated the adjusted prevalence of clinically important breathlessness to be 6.6% (95% CI = 6.5–6.7), after adjusting for age, sex, smoking status, place of residence, and educational attainment (Table S8 in the **Online Supplementary Document**). The multivariable model was statistically significant (Wald *F* = 3.21, *P* = 0.002). Sex and smoking status were independently associated with higher odds of breathlessness, while age, educational attainment, and place of residence were not.

### Breathlessness attribution

The three most commonly self-attributed causes of clinically important breathlessness were lung conditions other than tuberculosis (23.8%), ‘other’ causes (16.4%), and anaemia (11.6%). Respondents with mild breathlessness most commonly attributed it to lung conditions other than tuberculosis (26.6%), followed by anaemia (14.4%) and heart conditions (12.2%).

### Breathlessness impact

#### Quality of life

Respondents with clinically important breathlessness reported having poorer quality of life, with higher proportions reporting ‘moderate-to-extreme problems’ across all EQ-5D-5L dimensions (**Figure 1****,** **Table 2**). Respondents with an mMRC score of 3–4 reported fewer ‘moderate’ but more ‘severe or extreme’ problems than those with an mMRC score of 2 across all five dimensions. Respondents with mild breathlessness (mMRC 1) also reported poorer quality of life than those without breathlessness (mMRC 0).

**Figure 1:**
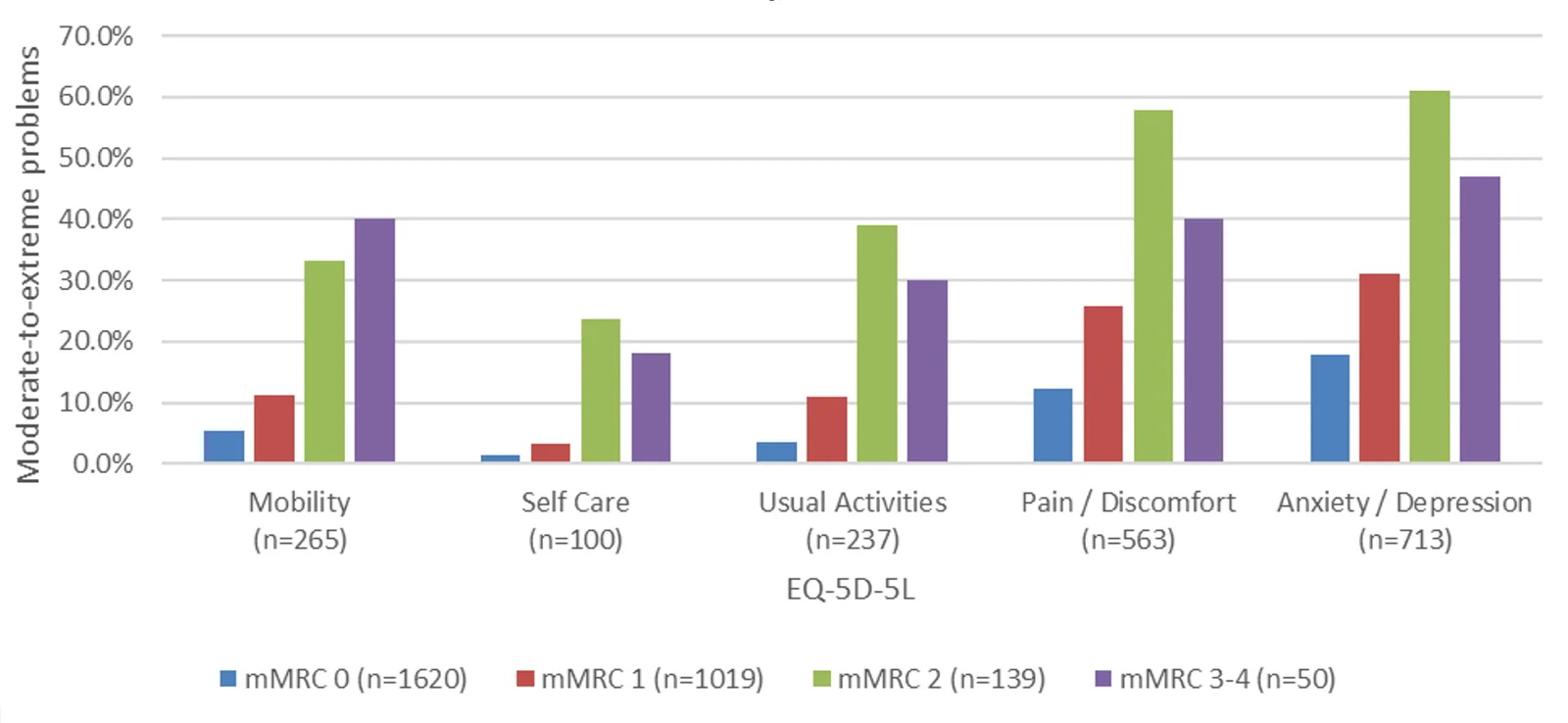
Proportion of moderate-to-extreme problems across EQ-5D-5L dimensions by level of breathlessness, measured using the mMRC breathlessness scale, among 2828 community-dwelling adults in South Africa (weighted data). EQ-5D-5L – EuroQol EQ-5D-5L descriptive system, mMRC – modified Medical Research Council breathlessness scale.

**Table 2:** Quality of life by breathlessness and disability by breathlessness level per the mMRC categories for respondents in an online survey in South Africa, weighted data*

	0 (n = 1,620, 57.3%)	1 (n = 1,019, 36.0%)	2 (n = 138, 4.9%)	3–4 (n = 50, 1.8%)	Total (n = 2,828, 100%)
**EQ-5D-5L and EQ-VAS**					
EQ-VAS score, x̄ (SD)	81.9 (18.8)	72.5 (20.1)	58.5 (20.7)	64.5 (30.3)	77.0 (20.6)
Mobility					
*I have no problems in walking about*	1,349 (83.3)	663 (65.1)	40 (29.2)	23 (46.9)	2,075 (73.5)
*I have slight problems in walking about*	185 (11.4)	243 (23.8)	52 (38.0)	7 (14.3)	487 (17.2)
*I have moderate problems in walking about*	60 (3.7)	100 (9.8)	30 (21.9)	11 (22.4)	201 (7.1)
*I have severe problems in walking about*	16 (1.0)	13 (1.3)	14 (10.2)	8 (16.3)	51 (1.8)
*I am unable to walk about*	10 (0.6)	0 (0.0)	1 (0.7)	0 (0.0)	11 (0.4)
Self-care					
*I have no problems washing or dressing myself*	1,522 (93.9)	868 (85.2)	77 (55.4)	33 (64.7)	2,500 (88.3)
*I have slight problems washing or dressing myself*	76 (4.7)	117 (11.5)	29 (20.9)	8 (15.7)	230 (8.1)
*I have moderate problems washing or dressing myself*	19 (1.2)	30 (2.9)	32 (23.0)	8 (15.7)	89 (3.1)
*I have severe problems washing or dressing myself*	3 (0.2)	3 (0.3)	1 (0.7)	1 (2.0)	8 (0.3)
*I am unable to wash or dress myself*	1 (0.1)	1 (0.1)	0 (0.0)	1 (2.0)	3 (0.1)
Usual activity					
*I have no problems doing my usual activities*	1,231 (76.0)	524 (51.4)	27 (19.4)	24 (48.0)	1,806 (63.9)
*I have slight problems doing my usual activities*	332 (20.5)	385 (37.8)	57 (41.0)	11 (22.0)	785 (27.8)
*I have moderate problems doing my usual activities*	51 (3.1)	95 (9.3)	48 (34.5)	7 (14.0)	201 (7.1)
*I have severe problems doing my usual activities*	5 (0.3)	14 (1.4)	6 (4.3)	7 (14.0)	32 (1.1)
*I am unable to do my usual activities*	1 (0.1)	1 (0.1)	1 (0.7)	1 (2.0)	4 (0.1)
Pain/discomfort					
*I have no pain or discomfort*	740 (45.7)	242 (23.7)	8 (5.8)	19 (38.0)	1,009 (35.7)
*I have slight pain or discomfort*	680 (42.0)	514 (50.4)	51 (36.7)	12 (24.0)	1,257 (44.4)
*I have moderate pain or discomfort*	166 (10.2)	210 (20.6)	59 (42.4)	9 (18.0)	444 (15.7)
*I have severe pain or discomfort*	27 (1.7)	45 (4.4)	9 (6.5)	8 (16.0)	89 (3.1)
*I have extreme pain or discomfort*	7 (0.4)	8 (0.8)	12 (8.6)	2 (4.0)	29 (1.0)
Anxiety/depression					
*I am not anxious or depressed*	840 (51.8)	343 (33.7)	18 (12.9)	17 (34.0)	1,218 (43.1)
*I am slightly anxious or depressed*	492 (30.4)	359 (35.2)	36 (25.9)	9 (18.0)	896 (31.7)
*I am moderately anxious or depressed*	201 (12.4)	213 (20.9)	53 (38.1)	8 (16.0)	475 (16.8)
*I am severely anxious or depressed*	66 (4.1)	67 (6.6)	17 (12.2)	6 (12.0)	156 (5.5)
*I am extremely anxious or depressed*	22 (1.4)	37 (3.6)	15 (10.8)	10 (20.0)	84 (3.0)
**WHODAS-12**					
Total score, x̄ (SD)	10.2 (8.5)	12.2 (8.3)	18.3 (8.9)	19.9 (10.1)	11.5 (8.8)
Total score, Mdn (minimum–maximum)	8 (0–38)	11 (0–39)	11 (0–39)	22 (0–48)	10 (0–48)
*‘Overall, in the past 30 days, how many days were these difficulties present?’, x̄ (SD)*	8.1 (8.1)	10.5 (9.1)	15.2 (8.7)	13.7 (10.5)	9.4 (8.7)
*‘Overall, in the past 30 days, how many days were these difficulties present?’, Mdn (minimum–maximum)*	5 (1–30)	7 (1–30)	15 (1–30)	10 (1–30)	5 (1–30)
*‘In the past 30 days, for how many days were you totally unable to carry out your usual activities or work because of any health condition?’, x̄ (SD)*	2.6 (4.2)	3.3 (4.8)	5.6 (5.3)	6.4 (6.5)	3.1 (4.6);
*‘In the past 30 days, for how many days were you totally unable to carry out your usual activities or work because of any health condition?’, Mdn (minimum–maximum)*	1 (0–29)	2 (0–28)	5 (0–25)	4 (0–25)	1 (0–29)
*‘In the past 30 days, not counting the days that you were totally unable, for how many days did you cut back or reduce your usual activities or work because of any health condition?’, x̄ (SD)*	2.6 (3.9)	3.5 (4.6)	5.1 (4.5)	5.7 (6)	3.1 (4.3)
*‘In the past 30 days, not counting the days that you were totally unable, for how many days did you cut back or reduce your usual activities or work because of any health condition?’, Mdn (minimum–maximum)*	1 (0–29)	2 (0–29)	5 (0–20)	4 (0–25)	2 (0–29)

EQ-5D-5L – EuroQol EQ-5D-5L descriptive system, EQ-VAS – EuroQol visual analogue scale, Mdn – median, mMRC – modified Medical Research Council breathlessness scale, SD – standard deviation, WHODAS-12 – World Health Organization Disability Assessment Schedule 2.0 12-item, x̄ – mean

*Presented as n (%) unless specified otherwise. The data have been rounded, so the total may not precisely match the individual values due to weighting.

The overall mean EQ-VAS score was 77.0 (SD = 20.6). Respondents reported poorer perceived health as breathlessness severity increased. After adjustment for age, sex, smoking status, educational attainment, and place of residence, differences in mean scores remained across breathlessness categories (Table S7 in the **Online Supplementary Document**). The adjusted mean score was 79.9 (95% CI = 77.4–82.3) among participants with an mMRC score of 0, 71.0 (95% CI = 68.2–73.7) among those with an mMRC score of 1, 57.0 (95% CI = 52.3–61.8) among those with an mMRC score of 2, and 63.6 (95% CI = 58.0–74.2) among those with mMRC scores of 3–4.

#### Disability

The mean WHODAS-12 was 11.5 (SD = 8.8). Respondents with clinically important breathlessness had higher scores than those with mild or no breathlessness (**Table 2**). After adjustment for age, sex, smoking status, educational attainment, and place of residence, differences in mean scores remained across breathlessness categories (Table S7 in the **Online Supplementary Document**). The adjusted mean score was 22.8 (95% CI = 21.8–23.8) among participants with an mMRC score of 0, 25.2 (95% CI = 24.0–26.4) among those with an mMRC score of 1, 31.4 (95% CI = 29.2–33.7) among those with an mMRC score of 2, and 32.4 (95% CI = 28.9-36.0) among those with mMRC scores of 3–4.

Mobility was the most affected disability domain among respondents with clinically important breathlessness, followed by participation (**Figure 2**). Among respondents with mild breathlessness, mobility and participation were the most affected domains, to the same extent.

**Figure 2:**
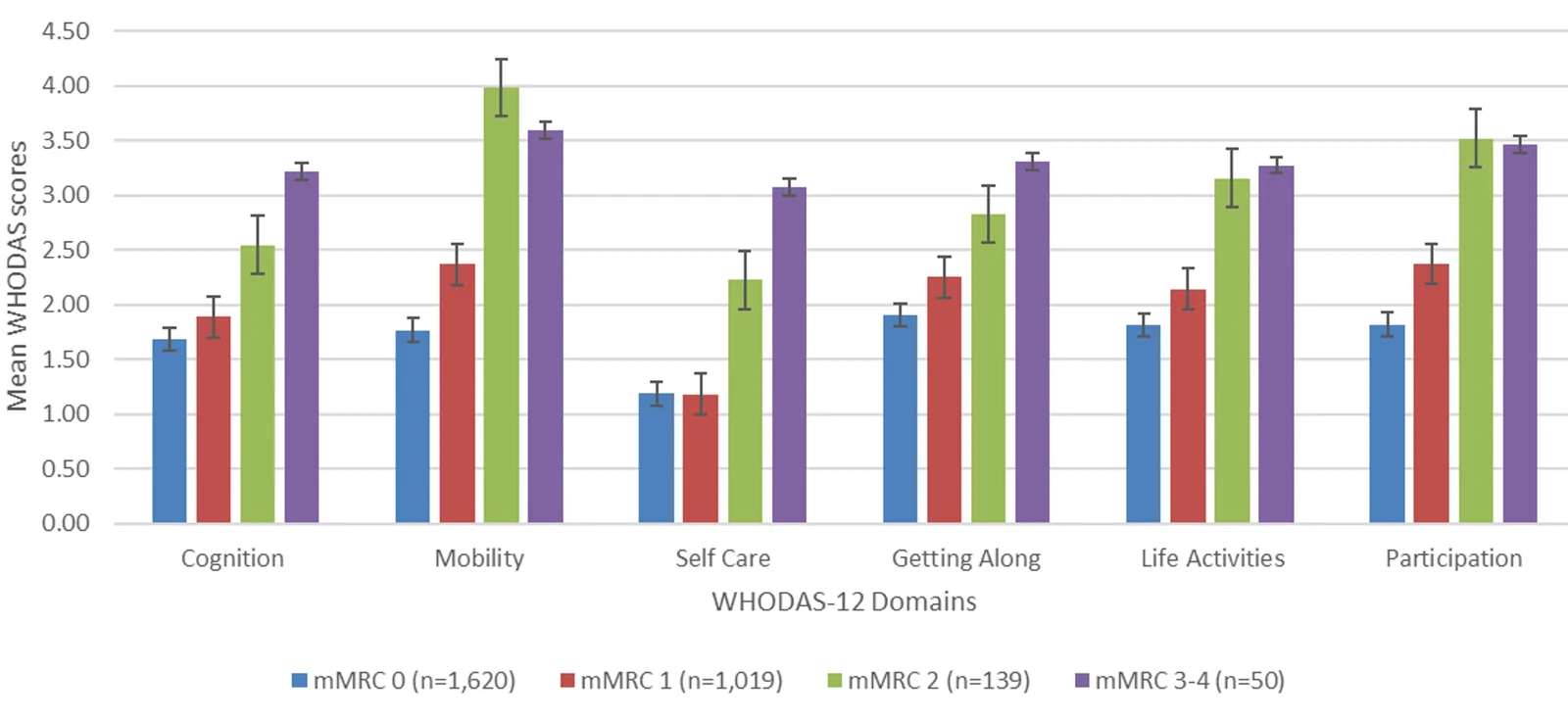
Mean disability scores with 95% CIs across WHODAS-12 domains by level of breathlessness, measured using the mMRC breathlessness scale, among 2,828 community-dwelling adults in South Africa (weighted data). CI – confidence interval, mMRC – modified Medical Research Council breathlessness scale, WHODAS-12 – World Health Organization Disability Assessment Schedule 2.0 12-item scale.

Respondents with mild breathlessness experienced disability for a mean of seven days per month, compared with 15 days among those with moderate (mMRC 2) breathlessness and 10 days among those with severe (mMRC 3–4) breathlessness (**Table 2**). Respondents with mild breathlessness reported reduced or stopped activities on a mean of two days per month, compared with five days among those with moderate breathlessness and four days among those with severe breathlessness.

#### Employment

Impacts on employment were reported by 64 of 189 (33.8%) respondents with clinically important breathlessness and 127 of 1,019 (12.5%) respondents with mild breathlessness (**Table 3**). Most of these respondents had worked full time before breathlessness affected their employment: 49 of 64 respondents with clinically important breathlessness (76.6%) and 87 of 127 respondents with mild breathlessness (68.5%).

**Table 3:** Self-reported impact of breathlessness on employment and activities of daily living of respondents in an online survey in South Africa (n = 1,208) (weighted data)*†

	mMRC			
	**1, n = 1,019 (84.4)**	**2, n = 139 (11.5)**	**3-4, n = 50 (4.1)**	**Total (n = 1,208)**
**Has your breathlessness affected your ability to work**				
Yes	127 (12.5)	54 (38.8)	10 (20.0)	191 (15.8)
No	861 (84.5)	77 (55.4)	40 (80.0)	978 (81.0)
Not applicable	30 (2.9)	7 (5.0)	0 (0)	37 (3.1)
**If yes, what was your previous employment status? (n = 191)**				
Worked full time	87 (68.0)	43 (79.6)	6 (60.0)	136 (70.8)
Worked less than full time	13 (10.2)	8 (14.8)	0 (0)	21 (10.9)
Worked on a casual basis	19 (14.8)	2 (3.7)	1 (10.0)	22 (11.5)
Other	8 (6.3)	0 (0)	3 (30.0)	11 (5.7)
Not applicable	1 (0.8)	1 (1.9)	0 (0)	2 (1.0)
**How much does your breathing affect you in your normal activities of daily living? *P* < 0.001**				
A lot	36 (3.5)	24 (17.3)	11 (22.0)	71 (5.9)
A little	615 (60.4)	104 (74.8)	32 (64.0)	751 (62.2)
Not at all	368 (36.1)	11 (7.9)	8 (16.0)	387 (32.0)

mMRC – modified Medical Research Council

*Presented as n (%).

†Data were self-reported by the respondents. Breathlessness in relation to exertion in daily life was assessed using the modified Medical Research Council (mMRC) breathlessness scale. The data have been rounded, so the total may not precisely match each number due to weighting.

#### Activities of daily living

Among respondents with clinically important breathlessness, 171 of 189 (90.3%) reported that the symptom had affected their everyday activities ‘a lot’ or ‘a little’. Among respondents with mild breathlessness, 651 of 1,019 (63.9%) also reported an impact on their everyday activities (**Table 3**).

### Sensitivity analyses

Findings were consistent across multiple sensitivity analyses. Estimates of breathlessness prevalence and associated outcomes were similar in the weighted and unweighted analyses (Table S7 in the **Online Supplementary Document**). Restricting the analyses to strata adequately represented in both the sample and population benchmarks yielded a breathlessness prevalence estimate of 6.6%, comparable to that obtained from the full weighted sample. These strata were defined by age, sex, and place of residence. More restrictive analyses incorporating strata defined by age, sex, and population group, where available, produced consistent results, although with reduced precision because of smaller sample sizes. These findings further supported the decision not to include province in the weighting model, as urban-rural residence captured broad geographic variation and the exclusion of province did not affect the estimated outcomes.

## DISCUSSION

Through this exploratory study, we found that approximately 7% of South African adults aged 18 years or older live with clinically important breathlessness (mMRC ≥ 2), corresponding to an estimated 4.4 million people living with moderate-to-severe breathlessness. A further 36% of adults, which would translate to approximately 22.7 million people, reported mild breathlessness (mMRC 1). Breathlessness was more common among women and was associated with poorer quality of life, greater disability, and adverse impacts on employment and daily functioning. These impacts increased with breathlessness severity.

A recent meta-analysis, which used different cut-off points to define breathlessness, estimated a pooled prevalence of 10% (95% CI = 7–15) in general adult populations, predominantly in high-income countries [4]. While direct comparisons are difficult because the studies in the aforementioned meta-analysis used different definitions of breathlessness, we found a slightly lower prevalence of clinically important breathlessness, at approximately 7%. Of note, our study population was also younger, with a mean age of 37 years, while most studies in the meta-analysis included populations with mean ages of 50 years or older. The higher prevalence of breathlessness among women found in our study is consistent with previous findings that activity-related breathlessness is twice as common among women as among men [36]. Physiological factors, such as reduced lung volumes, may partly explain this difference [36], as may differences in symptom perception and reporting [37–39]. However, studies have found that sex differences in breathlessness reporting disappeared after adjustment for mood (defined as positive affect and negative affect) [40,41]. Further research should investigate the factors underlying the higher reported prevalence of breathlessness among women in South Africa.

The most commonly self-attributed causes of clinically important breathlessness in South Africa were lung conditions other than tuberculosis, followed by ‘other’ causes and anaemia. The high prevalence of respiratory conditions other than tuberculosis, such as emphysema and bronchitis, is consistent with findings from the Burden of Obstructive Disease (BOLD) study, which reported that South Africa had the highest historical and current prevalence of chronic airflow obstruction among 40 participating countries [42]. Anaemia may contribute to breathlessness by reducing the blood’s oxygen-carrying capacity [43], and it disproportionately affects women in South Africa [44]. Future research should examine why the respondents frequently selected ‘other’ as an explanation of causes for breathlessness, as this response may reflect undiagnosed conditions or non-medical factors [45,46]. Overall, our findings suggest that multiple factors contribute to breathlessness in South Africa and may influence its clinical recognition and public health response.

Our findings highlight the wide-ranging effects of breathlessness on individual well-being, including greater disability and poorer quality of life across physical, psychosocial, and self-care domains. While disability increased with each level of breathlessness, which is consistent with previous findings [30], quality of life showed a more complex pattern when we compared severe breathlessness (mMRC 3–4) with moderate breathlessness (mMRC 2), with fewer ‘moderate’ and more ‘severe/extreme’ problems reported, respectively. This pattern may reflect a response shift, whereby people recalibrate their internal standards or reprioritise life domains as symptoms worsen. These changes may lead to more favourable self-ratings, despite objectively poorer functioning. The pattern may also reflect adaptation to worsening breathlessness, as people may have already reduced strenuous activities and therefore encounter fewer situations that provoke symptoms in daily life. Methodological factors may also have contributed, particularly the small number of respondents with severe breathlessness, especially those with an mMRC score of 4. Qualitative studies should examine whether these or other factors influence how people report quality of life and disability. Breathlessness also substantially disrupted daily activities, which is consistent with previous evidence [6]. These findings support the need for prompt, proactive, and ongoing clinical assessment of breathlessness, regardless of its aetiology.

Our findings are consistent with previous evidence that clinically important breathlessness may act as a barrier to paid employment [47]. This impact may have serious financial consequences for entire households, particularly for people from socioeconomically disadvantaged backgrounds [48]. People in South Africa may be unable to access financial support through a disability grant when breathlessness substantially limits their ability to work, but they do not have a formal diagnosis from a medical doctor. Breathlessness may therefore be linked to poverty, with consequences at both the individual and community levels [49]. Further research should examine this potentially complex and bidirectional relationship.

### Strengths and limitations

Here, we present preliminary data on the prevalence and impact of breathlessness among community-dwelling adults in South Africa. We also demonstrate the feasibility of collecting population-level symptom data independently of healthcare contact through an online survey with stratified sampling.

The survey’s web-based delivery may have enabled participation among people who were concerned about breathlessness, but were uncertain about, reluctant to seek, or unable to access clinical services. However, the web-based format may also have contributed to the higher proportion of respondents with greater educational attainment and may have underrepresented people with lower socioeconomic status. People from more disadvantaged groups may be more likely to experience long-term breathlessness, so the prevalence and impacts reported here may be underestimated. High levels of internet and mobile phone use in South Africa may have reduced potential digital bias [21]. Weighting partially corrected the higher educational attainment in the sample, although it remained substantially higher than that reported in the 2022 National Census. The survey was also conducted only in English, which may have further limited participation in this linguistically diverse country. Furthermore, we could not clinically verify the study measures, including self-reported attribution of breathlessness to an underlying condition. The survey also did not collect information on comorbidities or a history of treated pulmonary tuberculosis. Previous tuberculosis may be underreported in questionnaires [50]. The London Chest Activities of Daily Living item used to assess the impact of breathlessness on daily activities has not been validated as a standalone question, which limits comparisons with other studies.

### Implications for research, policy, and practice

Future research should investigate the extent of breathlessness in more rural, socially disadvantaged, and less English-proficient communities in South Africa using both cross-sectional and longitudinal designs. These studies should also examine potential financial and lifestyle effects, given the association between breathlessness and loss of employment observed in this study and the links between breathlessness and financial stress reported across different socioeconomic settings [51]. As breathlessness often remains unrecognised in routine care [52–54], the prevalence and impacts identified here support the need for timely and proactive recognition and assessment of chronic breathlessness.

South Africa is currently reforming its health system through the implementation of the National Health Insurance policy [55]. The policy aims to integrate disability and rehabilitation services fully into primary healthcare to improve access to care, treatment, and rehabilitation [56]. Our findings may help inform age- and context-appropriate assessment of breathlessness as part of routine care for this at-risk population.

## CONCLUSIONS

In this exploratory study, we estimated that more than 4 million people in South Africa live with moderate-to-severe breathlessness. Breathlessness was associated with poorer quality of life, greater disability, reduced employment, and poorer daily functioning. These findings highlight the long-term and life-changing consequences of breathlessness among community-dwelling adults. Prospective longitudinal studies should examine the extent and trajectory of these impacts on individuals and society to help inform clinical assessment.

## Additional material


Online Supplementary Document

## Data Availability

**Data availability:** All data are included in the manuscript and its supplements. The survey used in the study is available on the Open Science Framework: https://osf.io/6ztcw/.
