# Supplementary material for: Prevalence and impacts of breathlessness in South Africa: an exploratory, cross-sectional population study
Source: J Glob Health. 2026 Oct 9;16:04229. doi: 10.7189/jogh.16.04229 (PMC13650972; doi:10.7189/jogh.16.04229)
Supplement: Supplementary file 1 [file jogh-16-04229-s001.pdf]

Supplement to: Kochovska S, Brunelli VB, Chang S, Kinchin I, Eckert DJ, Clark JD, Sandberg J, Ekström M, Currow DC, van Zyl-Smit RN. Prevalence and impacts of breathlessness in South Africa: an exploratory, cross-sectional population study. J Glob Health. 2026;16:04229.

Table S1. The modified Medical Research Council (mMRC) breathlessness scale used in the study [3].

Please choose the one best response to describe any breathlessness that you have.	
0	I only get breathless with strenuous exercise
1	I get short of breath when hurrying on the level or up a slight hill
2	I walk slower than people of the same age on the level because of breathlessness or I have to stop for breath when walking at my own pace on the level
3	I have to stop for breath after walking 100 metres or after a few minutes on the level
4	I am too breathless to leave the house *or* breathless when dressing or undressing

Table S2. The question about breathlessness duration used in the study. Only respondents with a modified Medical Research Council (mMRC) breathlessness scale level 1 or above (mMRC $\geq$1) were asked this question, with responses denoting the duration of breathlessness related to the respondent's reported level of mMRC.

How long have you been experiencing breathlessness as severe as this?<br>*Enter approximate months and/or years*	
Enter months	<free text>
Enter years	<free text>

Table S3. The question about respondents' self-attributed primary cause of breathlessness used in the study. Only respondents with a modified Medical Research Council (mMRC) breathlessness scale level 1 or above (mMRC ≥1) were asked this question.

What do you understand is causing your breathlessness?<br>(Primary condition only; please choose one response only)	
☐	Tuberculosis
☐	Other lung conditions (e.g. emphysema, bronchitis, asthma, bronchiectasis)
☐	HIV/AIDS
☐	Heart conditions (e.g. angina, ischaemic heart disease / coronary artery disease, valve disease, abnormal heart rhythms)
☐	Disorder of nerves or muscles (e.g. motor neurone disease, multiple sclerosis)
☐	Cancer
☐	Low blood count (anaemia; low iron)
☐	Poor nutrition
☐	Coronavirus (COVID-19)
☐	Other (please specify): <free text>
☐	Do not know

Table S4. The question assessing the degree to which breathlessness affected respondents' activities of daily living used in the study [26]. Only respondents with a modified Medical Research Council (mMRC) breathlessness scale level 1 or above (mMRC ≥1) were asked this question.

How much does your breathing affect you in your normal activities of daily living?	
☐	A lot
☐	A little
☐	Not at all

Table S5. The questions assessing whether breathlessness had affected respondents' ability to work and, if yes, their previous employment status before breathlessness intervened. Only respondents with a modified Medical Research Council (mMRC) breathlessness scale level 1 or above (mMRC ≥1) were asked this question.

Has your breathlessness affected your ability to work?	
☐	Yes *(please complete below)*
☐	No
☐	Not applicable
If yes, what was your previous employment status?	
☐	Worked full time
☐	Worked less than full time
☐	Worked on a casual basis
☐	Other (please specify) <free text>
☐	Not applicable

Table S6. Residual imbalance in weighted data.

Variable	Weighted (%)	Benchmark* (%)	Residual Imbalance~ (%)
**Age**			
18 -19	3.8	4.8	1.0
20-24	12.5	12.1	0.4
25-29	13.5	13.5	0.0
30-34	13.2	13.1	0.1
35-39	12.3	11.9	0.4
40-44	9.9	9.8	0.1
45-49	7.8	8.0	0.2
50-54	6.5	6.8	0.3
55-59	5.9	5.7	0.2
60-64	4.9	4.6	0.3
65-69	3.8	3.6	0.2
70-74	2.5	2.5	0.0
75+	3.2	3.6	0.4
**Sex~**			
Male	47.7	48.1	0.4
Female	52.3	51.9	0.4
**Place of residence**			
Urban	68.3	68.2	0.1
Rural	31.7	31.8	0.1
**Education**			
Less than matriculation#	3.1	48.9	45.8
Matriculation	52.6	37.6	15.0
Higher education	44.3	13.5	30.8

Statistics South Africa Census 2022; ~The absolute percentage-point difference between the weighted sample and the benchmark; #Matriculation=completed secondary education

Table S7. Sensitivity analysis (overlap restriction).

Analysis	Prevalence (95% CI)	EQ-VAS* mean	WHODAS** mean
**Unweighted**	6.7 (5.6-7.7)		
mMRC 0		81.9 (80.7-83.0)	22.2 (21.7-22.7)
mMRC 1		72.5 (70.9-74.0)	24.2 (23.5-24.9)
mMRC 2		58.5 (54.6-62.5)	30.3 (28.4-32.1)
mMRC 3-4		64.5 (54.7-74.3)	31.9 (28.9-35.0)
**Weighted**	6.6 (6.5–6.7)		
mMRC 0		79.9 (77.4–82.3)	22.8 (21.8–23.8)
mMRC 1		71.0 (68.2–73.7)	25.2 (24.0–26.4)
mMRC 2		57.0 (52.3–61.8)	31.4 (29.2–33.7)
mMRC 3-4		63.6 (58.0–74.2)	32.4 (28.9-36.0)
**Restricted**<br>(Age x Sex x Place of residence)	6.5 (6.4 – 6.6)		
mMRC 0		79.6 (76.9–82.4)	23.3 (22.3-24.4)
mMRC 1		70.4 (67.4–73.3)	25.9 (24.7-27.2)
mMRC 2		56.6 (51.7–61.5)	32.2 (30.0, 34.4)
mMRC 3-4		63.5 (52.9–74.1)	32.9 (29.4-36.5)

CI = Confidence Intervals
* EQ-VAS=EQ-5D-5L Visual Analogue Scale; **WHODAS = World Health Organisation Disability Assessment Schedule 2.0 12-item.

Table S8. Multivariable logistic regression (modified Medical Research Council [mMRC] breathlessness scale level 2 and above [mMRC ≥2]): adjusted prevalence.

**Variables**	Adjusted OR	95% CI	p-value
Age	1.00	(0.99, 1.02)	0.25
Male (vs Female)	0.63	(0.44, 0.91)	0.01
Smoking (ref: Never)			
Current	2.16	(1.48, 3.16)	<0.001
Former	1.62	(1.00, 2.60)	0.05
Highest level of Education (ref: Post-secondary)			
Completed secondary	0.91	(0.65, 1.28)	0.59
Some education	1.28	(0.35, 4.69)	0.71
Urban (vs Rural)	1.26	(0.79, 2.01)	0.32

OR = Odds Ratio; CI = Confidence Intervals

Adjusted prevalence (%) of moderate-to-severe breathlessness (mMRC ≥2) is 6.6% (95% CI 6.5%-6.7%).
